# Descriptive study of plant resources in the context of the ethnomedicinal relevance of indigenous flora: A case study from Toli Peer National Park, Azad Jammu and Kashmir, Pakistan

**DOI:** 10.1371/journal.pone.0171896

**Published:** 2017-02-13

**Authors:** Muhammad Shoaib Amjad, Mirza faisal Qaeem, Israr Ahmad, Sami Ullah Khan, Sunbal Khalil Chaudhari, Nafeesa Zahid Malik, Humaira Shaheen, Arshad Mehmood Khan

**Affiliations:** 1 Department of Botany, Women University of Azad Jammu & Kashmir, Bagh, Pakistan; 2 Department of Botany, PMAS-University of Arid Agriculture, Rawalpindi, Pakistan; 3 Department of Botany Mirpur University of Science & Technology, Mirpur, Pakistan; 4 Department of Biosciences, COMSAT institute of Information Technology, Islamabad, Pakistan; Oklahoma State University, UNITED STATES

## Abstract

**Background:**

This paper presents the first quantitative ethnobotanical study of the flora in Toli Peer National Park of Azad Jammu and Kashmir, Pakistan. Being a remote area, there is a strong dependence by local people on ethnobotanical practices. Thus, we attempted to record the folk uses of the native plants of the area with a view to acknowledging and documenting the ethnobotanical knowledge. The aims of the study were to compile an inventory of the medicinal plants in the study area and to record the methods by which herbal drugs were prepared and administered.

**Materials and methods:**

Information on the therapeutic properties of medicinal plants was collected from 64 local inhabitants and herbalists using open ended and semi-structured questionnaires over the period Aug 2013-Jul 2014. The data were recorded into a synoptic table comprising an ethnobotanical inventory of plants, the parts used, therapeutic indications and modes of application or administration. Different ethnobotanical indices i.e. relative frequencies of citation (RFC), relative importance (RI), use value (UV) and informant consensus factor (Fic), were calculated for each of the recorded medicinal plants. In addition, a correlation analysis was performed using SPSS ver. 16 to check the level of association between use value and relative frequency of citation.

**Results:**

A total of 121 species of medicinal plants belonging to 57 families and 98 genera were recorded. The study area was dominated by herbaceous species (48%) with leaves (41%) as the most exploited plant part. The Lamiaceae and Rosaceae (9% each) were the dominant families in the study area. Among different methods of preparation, the most frequently used method was decoction (26 species) of different plant parts followed by use as juice and powder (24 species each), paste (22 species), chewing (16 species), extract (11 species), infusion (10 species) and poultice (8 species). The maximum Informant consensus factor (Fic) value was for gastro-intestinal, parasitic and hepatobiliary complaints (0.90). *Berberis lycium Ajuga bracteosa*, *Prunella vulgaris*, *Adiantum capillus-veneris*, *Desmodium polycarpum*, *Pinus roxburgii*, *Albizia lebbeck*, *Cedrella serrata*, *Rosa brunonii*, *Punica granatum*, *Jasminum mesnyi* and *Zanthoxylum armatum* were the most valuable plants with the highest UV, RFC and relative importance values. The Pearson correlation coefficient between UV and RFC (0.881) reflects a significant positive correlation between the use value and relative frequency of citation. The coefficient of determination indicated that 77% of the variability in UV could be explained in terms of RFC.

**Conclusion:**

Systematic documentation of the medicinal plants in the Toli Peer National Park shows that the area is rich in plants with ethnomedicinal value and that the inhabitants of the area have significant knowledge about the use of such plants with herbal drugs commonly used to cure infirmities. The results of this study indicate that carrying out subsequent pharmacological and phytochemical investigations in this part of Pakistan could lead to new drug discoveries.

## Introduction

Ethnobotany describes the complete relationship between people and plants and explores both the traditional botanical knowledge of local people and how they exploit plants for a variety of purposes [1–2]. Ethnobotanical studies emphasize the dynamic relationships between botanical diversity and social and cultural systems [3–4] and ethnobotanists are increasingly focusing on the application of different quantitative and statistical approaches to understand and accumulate knowledge on valuable plants in certain communities [5].

Medicinal knowledge about plants is receiving increasing attention and is recognized as a valuable asset worldwide for health care practices and as a driver of the conservation of medicinal plants [6]. For example, ethnobotany and ethno-pharmacological knowledge is considered to be an integral part of the knowledge required for drug development. ‘Ethnomedicine’ deals with cultural interpretations of health, disease and illness with a focus on different healing practices or processes concerned with gaining good health [7]. Based on traditional reports about the use and efficacy of plant-derived medicines, various plants are being screened in order to search for their active ingredients which may be employed in the development of novel drugs. According to the FAO, in the last few decades the number of known medicinal plants now reaches up to 50,000 different species which is 18.9% of the total world flora [8]. Despite the fact that traditional ethnomedicinal approaches may be considered to be outdated in comparison with modern westernised approaches to health care, the WHO report estimates that about 80% of the population in developing countries depend upon herbal medicines for curing aliments [9].

In Pakistan, the remote mountainous regions support a diversity of flora, with about 1572 plant genera and 5521 species [10]. In the mid-1990s, about 84% of the Pakistani population was reliant on herbal medication but now this traditional knowledge is confined only to remote areas of the country, particularly the mountainous regions. As indigenous knowledge is dynamic and changes with time, generation, culture and resources the accurate documentation of this knowledge is both timely and necessary [11]. The indigenous knowledge about medicinal plants among indigenous communities has been reported from various parts of the world [12–17] including Pakistan [18–27]. However, all these studies adapted qualitative approaches to document ethnobotanical information [28–29], while the use of quantitative approaches can lead to better interpretation of ethnobotanical data.

Azad Jammu and Kashmir is a lush mountainous area characterized by its diverse climate, soil and habitat types. A number of endemic medicinal plants of Pakistan are restricted to this area, while previous studies in different parts of Azad Jammu and Kashmir have revealed that the people possess a unique culture and have rich traditional knowledge [1, 30–32]. Toli Peer National Park supports some of the richest biodiversity in Kashmir. Most of the population in this area is rural with a low literacy rate. People lack modern health facilities and hence are dependent upon natural resources, especially plants, for healthcare and to compensate for low incomes. However, ethno-pharmacological studies specifically targeting the Toli Peer National Park are lacking, as is the validation of traditional uses of this area’s native plant species. This may be because the area is topographically challenging, comprising hills and steep slopes which make it difficult to access for research studies. In order to address this information gap, we undertook the present study with the aims of (i) compiling a complete inventory of the flora of the study area, and (ii) documenting the indigenous medicinal knowledge of these plants along with their methods of preparation and the folk recipes used by local herbalists. In addition, we also undertook various quantitative analyses in order to produce and compare relevant ethnobotanical indices in order to explore relationships between plant frequency of occurrence and ethnomedicinal use.

## Materials and methods

### Study area (climate, geo-ethnography and socio-economic conditions)

**Fig 1:**
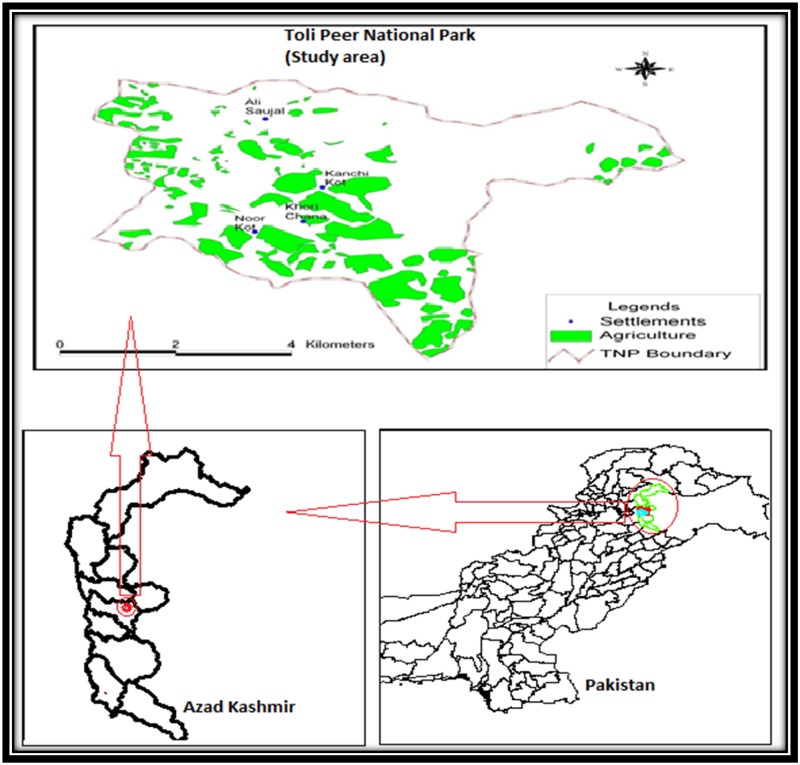
Map showing location of Toli Peer National Park within Pakistan and Azad Kashmir.

Toli Peer National Park is located in one of the world’s biodiversity hotspots. It is a mountainous area in Tehsil Rawalakot, District Poonch of Azad Kashmir, Pakistan. It lies at an altitude of 2546 m, with latitude 33.89°N and longitude 73.91°E. The climate of this region is of the moist temperate type. The maximum rainfall recorded is 1018 mm while the minimum is 3 mm during the summer monsoon in August and in October respectively. The average lowest temperatures are recorded in January (11C°) with temperature rising to maxima in June (average 34C°) [33–34]. There is heavy snow between November and March especially at higher elevations. The vegetation in the area comprises a wide variety of trees, shrubs, herbs, grasses and climbers with ground cover comprising a diversity of angiosperms along with ferns and mosses [33–34]. (A map of the study area is given in Fig 1).

A high proportion of the indigenous people of this hilly district are nomads. During the early summer months, they move their livestock herds from the plains to the higher mountainous areas of the National Park, and stay there for the whole of the summer season. Prior to the onset of winter, they make their way back down to the plains. A number of the main occupations are associated with summer tourism, including rest house managers, tour guides, shop keepers, restaurant workers and jeep drivers. However, many are full or part-time farmers and shepherds.

There is no formal marketing of medicinal plant in Toli Peer which by implication benefits home grown agents (middle man). Thus poor collectors have no share in high profit earning business. The study area was badly affected by an earthquake in 2005 which had a negative socioeconomic impact on the local population, including a rapid decline in the population sizes of some of the villages inside the National Park. The region is characterized by its remoteness, long distance from urban centers, difficult mountainous terrain, and a lack of government services, including modern health care facilities. As a result there is relatively high percentage of deaths among the more elderly members of the population as well as migration of many of the younger people away from the area to other safer and better developed centers. In the light of these demographic changes, it is vital to document the local knowledge of medicinal plant usage in this area before such information declines or is lost completely.

### Data collection

Field trips were conducted during Aug 2014-Jul 2015 in four seasons following the method of Heinrich and coworker [35]. During the study, 64 informants were selected randomly via convenience sampling of which 39 were males and 25 females. For the collection of ethnobotanical data, a semi-structured questionnaire was used to undertake one-on-one interviews in addition to group discussions [36–37] with some key informants as reported by Ghorbani *et al*. [19] The questionnaire was developed following the method of Edwards et al. [38] and required the informants to provide information regarding the local names of the medicinal plants, the diseases treated by herbal remedies, the plant parts used, the methods of preparation and the mode of administration. These discussions comprised both mixed as well as single gender discussions and were conducted in the local language, Pharari (Pothohari). The age of the informants ranged from 35 to 70 years. They included several *Hakeems* (traditional doctors) who were interviewed in order to record the local household recipes for the preparation of medicinal plants. Detailed demographic data are provided in Table 1. The informed consent from participants is also obtained to participate in this research before obtaining information. The permission for conducting research, field surveys and plant collection in Toli Peer national park was taken from chief conservator forest Department, Azad Jammu & Kashmir, Pakistan.

**Table 1:** Demographic data of informants in Toli Peer National park.

Variable	Categories	No. of Persons	Percentage
Informant category	Traditional healer	11	17.19
	Indigenous people	58	90.63
Gender	Female	25	39.06
	Male	39	60.94
Age	35–50 years	23	35.94
	50–65 years	28	43.75
	More than 65 years	18	28.13
Education Level	Illiterate	21	32.81
	Completed five years of education	16	25.00
	Completed eight years of education	11	17.19
	Completed 10 years of education	8	12.50
	Completed 12 years of education	7	10.94
	Some undergraduate (16 year education)	4	6.25
	Graduate (Higher education)	2	3.13
Experience of the traditional health practitioners	Less than 2 years	2	18.18
	2–5 years	4	36.36
	5–10 years	3	27.27
	More than 20 years	2	18.18

### Collection and identification of plants

Those plants in the study area that were identified as having a medicinal value were collected, pressed until dry, sprayed with a preservative 1% HgCl_2_ solution and mounted on to herbarium sheets. Voucher specimens were gathered and prepared according to standard taxonomic methods recommended by Jain and Rao [39]. For taxonomic identification, the Flora of Pakistan (www.eflora.com) was followed [40–41], whereas the International Plant Name Index (IPNI) (www.ipni.org) was used to obtain botanical names. The confirmation of identified plant was done in the Herbarium of Pakistan (ISL) Quaid—i–Azam University Islamabad, Pakistan. The fully determined vouchers were deposited in the herbarium of the Department of Botany, PMAS- Arid Agriculture University Rawalpindi, Pakistan.

### Quantitative ethnobotanical data analysis

For the validation and to test the homogeneity of the collected ethnobotanical data various quantitative indices were applied including use value (UV), relative frequency of citation (RFC), the informant consensus factor (Fic), and relative importance (RI). Association between indices was tested using correlation analysis.

#### Informant consensus factor (Fic)

The informant consensus factor was derived in order to seek an agreement between the informants on the reported cures for each group of diseases [42].

$$Fic=\frac{Nur\;–\;Nt}{\left(Nur\;–\;1\right)}$$

Where *Nur* is the number of use-reports in each disease category; *Nt* is number of species used.

#### Relative frequency of citation (RFC)

The index of relative frequency of citation (RFC) was determined by using the following formula [43]

$$\text{RFC}=\frac{\text{FC}}{\text{N}}$$

Where FC is the number of informants reporting use of a particular species and N is the total number of informants.

#### Use value index

The use value was calculated by using the following formula [43].

$$\text{UV}=\frac{\sum \text{Ui }}{\text{N}}$$

where Ui is the number of uses mentioned by each informant for a given species and N is the total number of informants.

#### Relative importance

The relative importance was calculated by applying the following formula [44].

$$\text{RI}=\left(\text{Rel PH }+\text{ Rel BS}\right)\times \frac{100}{2}$$

where PH is the pharmacological property of the given plant and Rel PH is the relative number of pharmacological properties ascribed to a single plant.

$$\text{Rel PH}=\frac{\text{PH of a given Plant }}{\text{Maximum PH of all reported plant species }}$$

BS is the number of body systems treated by a single species and Rel BS is the relative number of body systems treated by a single species

$$\text{Rel BS}=\frac{\text{BS of a given Plant}}{\text{Maximum BS of all reported plant species }}$$

#### Jaccard index (JI)

To compare the study with already published work and to access similarity of knowledge among different communities, the Jaccard index [45] was calculated using the following formula

$$\text{JI}=\frac{\text{c}\times 100}{\left(a+b\right)-c}$$

Where “a” is the number of species of the area A (our study area); “b” is the number of species of the neighboring area B; and “c” is the number of species common to both A and B.

#### Pearson correlation

Pearson Correlation analysis was carried out between the RFC and UV using SPSS ver. 16, the r^2^ was also calculated to measure cross species variability in RFC explained by variance in UV.

## Results and discussion

### Family contribution and habit of ethnomedicinal flora

**Fig 2:**
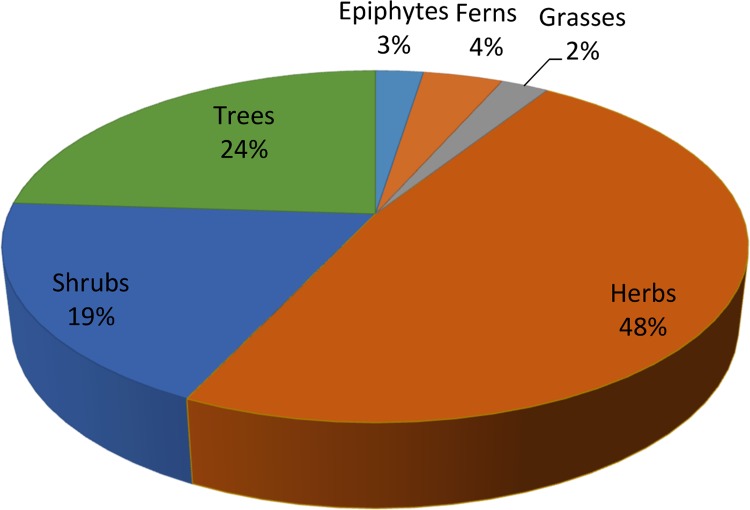
Life form contribution of ethnomedicinal-flora.

Altogether 121 medicinal plant species belonging to 98 genera and 57 families are reported (Table 2). Lamiaceae and Rosaceae (11 species each) are the dominant families of the study area followed by Asteraceae (10 species), Papilionaceae (6 species) and Ranunculaceae (6 species). The remaining families contribute ≤5 species in the ethnomedicinal flora of the study area. The dominance of these families is attributed to the fact that they are abundant in the area and easily available to the local people. In addition, people of the area have a high knowledge about plants from these families, i.e. they have been using these plants for many generations and hence the members of these plant families are well known to them. This is probably due to the presence of secondary metabolites in important plant species of these families. A similar report was presented earlier by [46] where Lamiaceae, Moraceae, Astraceae, Mimosaceae, Apocyanaceae and Liliaceae were documented as dominant ethnomedicinal plant families among a total of 25 families from Darra Adam Khel NWFP, Pakistan. The majority of the medicinal plant species identified in the study area are reportedly utilized to treat respiratory disorders, followed by gastrointestinal and other complaints (Tables 3 and 4). This result is also in agreement with previous studies. For example, Abbasi *et al*. [47] reported 89 ethnomedicinal plant species in 46 families from the Lesser Himalayas of Pakistan with the highest informant consensus factor reported for pathologies related to respiratory and reproductive disorders. Similarly, Kiyani *et al*. [48] reported use of 120 plant species from 51 plant families that were applied in the treatment of 25 different respiratory problems by the inhabitants of Gallies-Abbottaba in northern Pakistan. There is a particular prevalence of respiratory diseases in the study area due to the high altitude combined with low barometric pressure which limits the supply of oxygen (O_2_) thereby impacting on lung function [49]. Most of the plant species in the area identified as having an ethnomedicinal value were herbaceous (58%), followed by trees (29%), shrubs (23%), ferns (5%), grasses (3%) and climbers (3%) (Fig 2). These results reflect the high altitude of the study area where the herbaceous flora is dominant with fewer shrubs and trees.

**Table 2:** Distribution of medicinal plant species according to their family.

Family	No. of Species	%age contribution	Family	No. of Species	%age contribution
Lamiaceae	11	9.09	Borangniceae	1	0.83
Rosaceae	11	9.09	Buxaceae	1	0.83
Asterceae	10	8.26	Companulaceae	1	0.83
Paplionaceae	6	4.96	Cucurbitaceae	1	0.83
Ranunculaceae	6	4.96	Dryopteridaceae	1	0.83
Fragaceae	5	4.13	Fumaricaceae	1	0.83
Adiantaceae	3	2.48	Gentianaceae	1	0.83
Apiaceae	3	2.48	Guttiferae	1	0.83
Caprifoliaceae	3	2.48	Hippocotanaceae	1	0.83
Pinaceae	3	2.48	Juglandaceae	1	0.83
Poaceae	3	2.48	Malvaceae	1	0.83
Dioscoreaceae	2	1.65	Melliaceae	1	0.83
Elaeagnaceae	2	1.65	Mimoaceae	1	0.83
Euphorbiaceae	2	1.65	Myrsinaceae	1	0.83
Liliaceae	2	1.65	Onagraceae	1	0.83
Moraceae	2	1.65	Plantaginaceae	1	0.83
Oleaceae	2	1.65	Podophyllaceae	1	0.83
Polygonoceae	2	1.65	Primulaceae	1	0.83
Rubicaceae	2	1.65	Pteridaceae	1	0.83
Rutaceae	2	1.65	Punicacea	1	0.83
Salicaceae	2	1.65	Rhamnaceae	1	0.83
Violaceae	2	1.65	Sambucaceae	1	0.83
Acanthaceae	1	0.83	Sapindaceae	1	0.83
Alliaceae	1	0.83	Saxifragaceae	1	0.83
Anacardiaceae	1	0.83	Smilicaceae	1	0.83
Apocynaceae	1	0.83	Ulmaceae	1	0.83
Araliaceae	1	0.83	Urticaceae	1	0.83
Asclepidaceae	1	0.83	Valerianaceae	1	0.83
Berberidaceae	1	0.83			

**Table 3:** Medicinal flora of Toli Peer National Park, Azad Jammu and Kashmir, Pakistan.

S#	Binomial /Voucher number	Local name	Habit	Part used	Method of preparation/property	Mode of application	Disease treated	Rel BS	Rel PH	RI	FC	RFC	UV
**Acanthaceae**													
1	*Dicliptera bupleuroides* Nees in Wall./mh-03	Kirch, somni, herb	Herb	Leaves	Paste	External	Wounds, eczema.	0.29	0.5	39.29	52	0.81	0.86
				Leaves	Decoction	External	Tonic, cough.						
**Adiantaceae**													
2	*Adiantum capillus-veneris* L./mh-04	Hansraj, Sraj fern	Fern	Leaves	Decoction	Internal	Boils, cough, asthma, jaundice, cold, diabetes, skin diseases, measles, eczema, chest pain	0.71	0.83	77.38	57	0.89	0.97
3	*Adiantum incisum* Foressk/mh-06	Sumbul, Hansraj fern	Fern	Leaves	Juice	Internal	Scabies, cough, fever, skin diseases	0.29	0.5	39.29	44	0.69	0.64
4	*Athyrium tenuifrons* Wall.apud Moore ex. R. Sim./mh– 07	Fern	Fern	Root	Tea	Internal	Body pain	0.14	0.33	23.81	32	0.5	0.58
				Root	Powder	External	Wounds						
**Alliaceae**													
5	*Allium griffithianum* Boiss./mh– 09	Piazi	Herb	Aerial parts	Cooked	Internal	Carminative, used in dyspepsia, flatulance and colic	0.29	0.17	22.62	29	0.45	0.53
**Anacardiaceae**													
6	*Pistacia chinensis* Bunge/mh -11	Kangar	Tree	Stem gum	Powder	Internal	Dysentery	0.21	0.33	27.38	43	0.67	0.91
				Bark	Paste	External	Wounds, cracked heels						
7	*Heracleum candicans* Wall ex. DC/mh -12	----	Herb	Aerial parts	Tea	Internal	Nerve disorders	0.07	0.17	11.9	12	0.19	0.14
8	*Pimpinella stewartii* Dunn. E. Nasir/mh-13	Tarpakki	Herb	Fruit	Eaten	Internal	Stomach disorder	0.07	0.17	11.9	12	0.19	0.3
**Apiaceae**													
9	*Heracleum cachemirica* C.B. Clarke/mh -14	Shrub	Shrub	Aerial parts	Juice	Internal	Nerve disorders	0.07	0.17	11.9	18	0.28	0.19
**Apocyanaceae**													
10	*Nerium oleander* Linn./mh -15	Kanair	Tree	Leave	Paste	External	Cutaneous eruption	0.57	0.67	61.9	46	0.72	0.98
				Leave	Decoction	Internal	Wounds and swelling						
				Bark	Decoction	Internal	Skin diseases, leprosy						
				Roots	Powder	Internal	Abortion						
				Roots	Paste	External	Scorpion sting, snake bite						
**Araliaceae**													
11	*Hedera nepalensis* K. Koch/mh -16	Harbumbal epiphyte	Epiphyte	Leaves	Decoction	Internal	Diabetes	0.07	0.17	11.9	11	0.17	0.13
**Asclepidaceae**													
12	*Vincetoxicum hirundinaria* Medicres/mh-17	----	Herb	Aerial parts	Decoction	Internal	Boils, pimples	0.14	0.17	15.48	48	0.75	0.8
**Asteraceae**													
13	*Anaphalis adnata* D.C/mh-18	----	Herb	Leaves	Powder	External	Bleeding cuts and wounds	0.14	0.17	15.48	19	0.3	0.42
14	*Artemisia absinthium* L./mh -19	Afsanthene	Herb	Leaves	Infusion, paste	Internal	Anthelmintic, stomach disorders, wounds and cuts	0.29	0.5	39.29	51	0.8	0.98
15	*Artemisia maritime* L./mh -21	Afsanthene	Herb	Leaves	Paste	External	Skin infections	0.14	0.33	23.81	41	0.64	0.77
				Leaf and stem	Powder	Internal	Intestinal parasites						
16	*Artemisia dubia* Wall./mh-22	Asfanthene	Herb	Seeds	Cooked	Internal	Weakness after delivery	0.36	0.67	51.19	23	0.36	0.52
				Leaves	Paste	External	Cuts and wounds, ear diseases						
				Aerial parts	Extract	External	Vermicide						
17	*Conyza bonariensis* L Cronquist/mh-24	Buti	Herb	Aerial parts	Infusion	Internal	Diarrhea and dysentery, bleeding piles	0.21	0.17	19.05	41	0.64	0.77
18	*Gerbera gossypina* (Royle) Beauverd/mh-25	Put potula	Herb	Aerial parts	Tea	Internal	Nerve disorders	0.07	0.17	11.9	12	0.19	0.14
19	*Parthenium hysterophorus* L./mh-27	Herb	Herb	Root	Decoction	Internal	Skin disorders, dysentery	0.14	0.33	23.81	35	0.55	0.59
20	*Saussurea candolleana* Wall. Ex. D.C Clarke/mh-29	Herb	Herb	Roots	Extract	Internal	Tonic	0.07	0.17	11.9	23	0.36	0.28
21	*Taraxacum officinale* F. H. Wigg/mh-31	Handh	Herb	Roots	Decoction	Internal	Jaundice	0.29	0.67	47.62	56	0.88	0.92
				Leaves	Cooked	Internal	Swellings, diuretic, tonic						
22	*Achillea millefolium* L./mh-32	Yarrow	Herb	Flower	Extract	Internal	Soft drinks	0.14	0.33	23.81	24	0.38	0.33
				Leaves	Powder	External	Toothache						
23	*Berberis lycium* Royl/mh-33	Sumblu	Shrub	Roots	Extract	Internal	Tonic, eye lotion, skin disease, chronic diarrhea, piles, blood purifier, diabetes, pustules, scabies	0.64	1.33	98.81	59	0.92	0.98
				Roots	Paste	External	Bone fracture						
**Boraginaceae**													
24	*Trichodesma indicum* L. R. Br/mh-35	Handusi booti	Herb	Leaves	Boiling	Internal	Flu and cough	0.14	0.17	15.48	31	0.48	0.48
**Buxaceae**													
25	*Sarcococca saligna* D. Don Muell/mh-37	Bansathra	Shrub	Leaves and shoots	Decoction	Internal	Joint pain, laxative, blood purifier	0.36	0.83	59.52	23	0.36	0.23
				Leaves	Powder	External	Burns						
				Root	Juice	Internal	Gonorrhoea						
**Caprifoliaceae**													
26	*Vibernum nervosum* D. Don/mh-39	Taliana	Shrub	Fruit	Eaten	Internal	Stomach ache, anemia	0.14	0.33	23.81	15	0.23	0.3
27	*Viburnum grandiflorum* Wall.ex DC/mh-40	Guch, shrub	Shrub	Seed	Juice	Internal	Typhoid, whooping cough	0.14	0.33	23.81	25	0.39	0.2
28	*Viburnum cotinifolium* D. Don/mh-41	Taliana	Shrub	Fruit	Eaten	Internal	Laxative, blood purifier	0.21	0.5	35.71	31	0.48	0.33
				Leaves	Extract	Internal	Menorrhagia						
**Companulaceae**													
29	*Campanula benthamii* Wall./mh-42	Herb	Herb	Root	Chewing, earache	External	Strengthen heart, earache	0.14	0.33	23.81	19	0.3	0.36
**Cucurbitaceae**													
30	*Momordica dioica* Roxb. ex Willd/mh-43	Epiphyte	Epiphyte	Roots	Cooked	Internal	Piles, urinary problem	0.14	0.33	23.81	15	0.23	0.17
**Dioscoreaceae**													
31	*Dioscorea bulbifera* L./mh-45	Herb	Herb	Aerial parts	Juice	Internal	Contraceptive	0.07	0.17	11.9	41	0.64	0.81
32	*Dioscorea deltoidea* Wall. ex Kunth/mh-47	Herb	Herb	Rhizome	Eaten	Internal	Insect killer, snake bite	0.14	0.33	23.81	36	0.56	0.48
**Dryopteridaceae**													
33	*Polystichum squarrosum* Don Fee/mh-49	Fern	Fern	Root	Decoction	Internal	Pyloric disease	0.07	0.17	11.9	13	0.2	0.3
**Elaeagnaceae**													
34	*Elaeagnus angustifolia* Linn./mh-50			Ripe fruits	Boiled	Internal	Sore throat, high fever	0.29	0.5	39.29	29	0.45	0.66
				Fruit	Eaten	Internal	Cough and cold						
35	*Elaeagnus umbellata* Thunb./mh-51	Russian olive, Tree		Leaves	Decoction	Internal	Cough	0.29	0.67	47.62	33	0.52	0.73
				Flowers	Decoction	Internal	Heart disease						
				Seeds	Eaten	Internal	Immunity						
				Branch	Exude	External	Toothache						
**Euphorbiaceae**													
36	*Euphorbia helioscopia* Linn./mh-53	Dhodhal, dandlion	Herb	Seeds	Juice	Internal	Cholera	0.14	0.17	15.48	49	0.77	0.72
				Roots	Paste	Internal	Anthelmintic						
37	*Euphorbia wallichii* Hk. f./mh-54	Dhodhal dandlion	Herb	Aerial parts	Latex	Internal	Laxative, purgative, digestive	0.36	0.33	34.52	42	0.66	0.91
				Aerial parts	Juice	Internal	Warts, skin infections						
**Fagaceae**													
38	*Castanea sativa* Mill./mh-56	Chest nut	Tree	Leaves	Infusion	Internal	Fevers	0.14	0.33	23.81	21	0.33	0.38
				Leaves	Decoction	Internal	Sore throats						
**Fabaceae**													
39	*Dalbergia sissoo* Roxb./mh-57	Tahli	Tree	Stem bark	Juice	External	Skin allergy	0.21	0.5	35.71	39	0.61	0.77
				Crushed leaves	Juice	Internal	Blood purifier						
				Leaves	Washing	External	Increase hair length						
**Fragaceae**													
40	*Quercus baloot* Griff/mh-59	Rein, Shah baloot, Oak	Tree	Bark	Powder	Internal	Asthma	0.29	0.33	30.95	43	0.67	0.86
				Nut	Decoction	Internal	Urinary problems, cough, cold						
41	*Quercus dilatata* Royle/mh-62	Oak, barungi	Tree	Fruit	Powder	Internal	Tonic	0.14	0.33	23.81	47	0.73	0.36
				Bark	Decoction	Internal	Dysentery						
42	*Quercus incana* Roxb./mh-64	Rein, ban, rinji	Tree	Bark	Powder	Internal	Asthma, cough, fever, rheumatism and backache	0.36	0.5	42.86	41	0.64	0.95
**Fumaricaceae**													
43	*Fumaria indica* (Hausskan) Pugsley/mh-66	Papra	Herb	Aerial parts	Juice, paste	Internal	Fever, constipation, pimples, eruption, skin infections, purify blood	0.43	0.67	54.76	48	0.75	0.84
**Gentianaceae**													
44	*Swertia ciliate* G. Don B. L. Burtt/mh-67	Herb	Herb	Aerial part	Decoction	Internal	Cough cold and fever	0.21	0.33	27.38	48	0.75	0.88
**Guttiferae**													
45	*Hypericum perforatum* L./mh-68	Herb	Herb	Flowers	Infusion	Internal	Snake bite wounds, sores, swellings, ulcers, rheumatism	0.36	0.5	42.86	47	0.73	0.61
**Hippocotanaceae**													
46	*Aesculus indica* (Wall. Ex Camb.) Hook.f.)/mh-69	Bankhore, horsechestnut	Tree	Bark	Infusion	Internal	Tonic	0.29	0.67	47.62	33	0.52	0.5
				Fruits	Eaten	Internal	Colic, rheumatic pains						
				Seed	Powder	Internal	Leucorrhoea						
**Juglandaceae**													
47	*Juglans regia* L./mh-70	Akhrot, khore	Tree	Leave	Decoction	External	Antispasmodic	0.36	0.67	51.19	51	0.8	0.92
				Bark	Rubbing	External	Gums and cleaning teeth, make lips and gums dye						
				Seeds	Oil	External	Rheumatic pain						
				Roots and leaves	Powder	External	Antiseptic						
**Lamiaceae**													
48	*Isodon rugosus* Wall. ex Benth. Codd./mh-72	Khwangere	Shrub	Leaves	Decoction	Internal	Blood pressure, toothache, body temperature, rheumatism	0.29	0.67	47.62	37	0.58	0.75
49	*Ajuga bracteosa* Wall, ex Benth/mh-73	Ratti booti	Herb	Aerial parts	Extract	Internal	Blood purification, body inflammation, eruption, pimples	0.64	1	82.14	58	0.91	1
				Leaves	Extract	Internal	Earache, eye ache, boils, mouth gums, throat pain						
50	*Nepeta erecta Royle* ex. Benth Benth/mh-75	Herb	Herb	Flowers	Juice	Internal	Cough	0.43	0.67	54.76	53	0.83	0.78
				Leaves	Juice	Internal	Blood pressure, cold, fever, influenza, toothache						
51	*Nepeta laevigata* D. Don Hand/mh-77	Herb	Herb	Fruit	Infusion	Internal	Dysentery	0.07	0.17	11.9	17	0.27	0.22
52	*Mentha royleana subsp*. *hymalaiensis* Briq./mh-79	Podina	Herb	Leaves	Juice, Powder to make chattni	Internal	Stomach disorder, gas trouble, indigestion, vomiting, cholera, fever and cough	0.5	0.5	50	58	0.91	0.97
53	*Prunella vulgaris* L./mh-81	Herb	Herb	Seeds	Eaten	Internal	Laxative, antipyretic, tonic, diuretic, inflammation, heart disease difficult breathing, eye sight weakness	0.57	1	78.57	58	0.91	0.98
54	*Salvia hians* Royle/mh-82	Herb	Herb	Leaves	Juice	Internal	Cough, colds, anxiety	0.21	0.33	27.38	31	0.48	0.66
55	*Salvia lanata* Roxb./mh-83	Herb	Herb	Leaves	Poultice	External	Skin problems, wounds	0.14	0.33	23.81	27	0.42	0.48
56	*Salvia moorcroftiana* Wall. Ex Benth/mh-84	Kaljari	Herb	Aerial parts	Juice	Internal	Diarrhea, gas trouble, stomach disorders, cough	0.29	0.33	30.95	51	0.8	0.89
57	*Thymus liniaris* Benth. ex Beth./mh/85	Herb	Herb	Leaves and flowers	Powder	Internal	Strengthen teeth, gum infection, bleeding	0.29	0.5	39.29	32	0.5	0.64
				Flower	Along ground seeds of *Carum carvi*	Internal	Improve digestion						
**Liliaceae**													
58	*Asparagus filicinus* Ham. in D. Don/mh-87	Herb	Herb	Root	Decoction	Internal	Diuretic, antipyretic, stomachic, nervous stimulant	0.29	0.5	39.29	38	0.59	0.66
59	*Polygonatum multiflorum* L. Smith/mh-88	Herb	Herb	Leave	Paste	External	Wounds	0.07	0.17	11.9	17	0.27	0.19
**Meliaceae**													
60	*Cedrella serrata* Royle/mh-89	Drawa	Tree	Stem and root bark	Paste	External	Round worms	0.5	1	75	54	0.84	0.83
				Leaves	Juice	Internal	Digestive problems, diabetes						
				Leaves	Decoction	External	Cooling agent, excellent hair washing						
				Bark	Poultice	Internal	Ulcers,						
				Bark	Powder	Internal	Chronic infantile dysentery						
**Mimosaceae**													
61	*Albizia lebbeck* Linn. (Benth)./mh-90	Shirin	Tree	Seeds	Powder	External	Inflammation, skin diseases, leprosy, leukoderma	1	0.5	75	57	0.89	0.83
				Bark	Powder	External	Strengthen spongy gums						
				Bark and seeds	Extract	Internal	Piles, diarrhea and dysentery						
				Flowers	Paste	External	Carbuncles, boils, swelling and other skin diseases						
				Seed	Oil	External	Snake bite, breathing problems						
**Malvaceae**													
62	*Malvastrum coromandelianum* Linn. (Garcke)/mh-91	Herb	Herb	Aerial parts	Decoction	Internal	Kill worms, dysentery	0.14	0.33	23.81	38	0.59	0.41
**Moraceae**													
63	*Ficus palmate* Forssk./mh-92	Phaghwar, anjir	Tree	Fruit	Eaten	Internal	Demulcent laxative, diseases of the lungs and the bladder, cooling agent, laxative	0.43	0.5	46.43	37	0.58	0.84
				Aerial parts	Paste	External	Freckles						
				Latex		External	Skin problem						
64	*Ficus carica* L/mh-94.	Phagwar	Tree	Fruit	Eaten	Internal	Constipation, piles, urinary bladder problems, anemia, constipation	0.57	0.67	61.9	52	0.81	0.95
				Leaves	Latex	External	Nail wound.						
				Latex	Rubbing	External	Extract spines from feet or other body organs						
**Myrsinaceae**													
65	*Myrsine africana* Linn./mh-95	Gorkhan, chapra, bebrang	Shrub	Fruits	Powder	Internal	Anthelmintic, carminative, stomach tonic, laxative	0.36	0.5	42.86	49	0.77	0.84
				Leaves	Decoction	Internal	Blood purifier						
**Oleaceae**													
66	*Jasminum mesnyi* Hance/mh-97	Pili chambali	Shrub	Leaves	Powder	External	Dandruff, muscular pains	0.5	0.83	66.67	51	0.8	0.67
				Leaves	Chewing	Internal	Mouth ulcers						
				Leaves	Decoction	Internal	Pyorrhea						
				Branches	Ash	External	Migraine and small joint pain						
				Dried flower	Powder	Internal	Hepatic disorders						
67	*Ligustrum lucidum* W. T. Aiton/mh-99	Guliston	Shrub	Aerial parts	Extracts	Internal	Antitumor	0.07	0.17	11.9	23	0.36	0.5
**Onagraceae**													
68	*Oenothera rosea* L.Her. ex Ait/mh-100	Buti	Herb	Leaves	Infusion	Internal	Hepatic pain, kidney disorders	0.14	0.33	23.81	45	0.7	0.64
**Paplionaceae**													
69	*Sophora mollis* Royle Baker/mh-101	Shrub	Shrub	Flowers	Powder	External	Pimples, sun burns, swellings, wounds	0.29	0.5	39.29	21	0.33	0.36
70	*Alysicarpus bupleurifolius* L. D.C/mh-102	Herb	Herb	Leaves	Juice	Internal	Blood purification.	0.07	0.17	11.9	15	0.23	0.22
71	*Melilotus alba* Desr/mh-104	Herb	Herb	Leaves	Paste	External	Joint inflammation	0.07	0.17	11.9	15	0.23	0.3
72	*Robinia pseudo-acacia* L./mh-105	Kikar	Tree	Bark	Chewing	External	Toothache	0.07	0.17	11.9	31	0.48	0.8
73	*Desmodium polycarpum* DC./mh-107	Shrub	Shrub	Roots	Juice	Internal	Fever, cardiac tonic, diuretic, loss of appetite, flatulence, diarrhea, dysentery, nausea, piles, helminthiasis, cough, fever	0.86	0.67	76.19	34	0.53	0.88
74	*Lespedeza juncea* Linn.f./mh-108	Herb	Herb	Root	Juice	Internal	Diarrhorea and dysentery	0.14	0.17	15.48	26	0.41	0.38
**Pinaceae**													
75	*Abies pindrow* Royle/mh-109	Partal, Paluder silver fir	Tree	Leaf	Paste	External	Swelling	0.57	0.67	61.9	48	0.75	1.03
					Juice	Internal	Fever						
				Bark	Powder	Internal	Cough, Chronic asthma						
				Bark	Tea	Internal	Rheumatism						
				Resin		External	Cuts and wounds						
				Root	Decoction	Internal	Cough, bronchitis						
76	*Pinus roxburgii* Roxb/mh-111	Chir	Tree	Leaves bark Powder	Juice	Internal	Dysentery	0.5	1	75	58	0.91	1.13
				Resin	Poultice	Internal	Ulcer, tumors, bleeding, wounds, severe cough, snake bite						
77	*Pinus wallichiana* A.B. Jackson/mh-112	Biar, blue pine	Tree	Resin	Poultice	External	Cuts and wounds	0.14	0.17	15.48	42	0.66	0.84
**Poaceae**													
78	*Desmostachya bipinnata* L. Stapf./ mh-115	Grass	Grass	Roots	Tea	Internal	Hypertension	0.07	0.17	11.9	14	0.22	0.17
79	*Poa nepalensis* Walls ex. Duthie./mh-117	Grass	Grass	Leaves	Decoction mixed with water	External	Anti lice	0.07	0.17	11.9	29	0.45	0.42
80	*Themeda ananthra* Nees ex Steud. Anderss./mh-118	Grass	Grass	Aerial parts	Poultice	External	Lumbago	0.14	0.33	23.81	41	0.64	0.5
				Leaves	Decoction	Internal	Blood purifier						
**Plantaginaceae**													
81	*Plantago lanceolata* L./mh-119	Ispgol	Herb	Leaves	Paste	External	Wounds	0.36	0.5	42.86	53	0.83	0.91
				Seeds	Extract	Internal	Tooth ache, dysentery, purgative, haemostatic						
**Podophyllaceae**													
82	*Podophyllum emodi* Wall ex Royle/mh-122	Banhakri	Herb	Root	Extract	Internal	Purgative, stomach diseases, liver and bile diseases	0.36	0.5	42.86	48	0.75	0.83
**Polygonoceae**													
83	*Rumex hastatus* L./mh-124	Khatimal	Shrub	Roots	Juice	Internal	Asthma, cough, and fever, weakness in cattle	0.29	0.5	39.29	32	0.5	0.64
84	*Rumex dentatus* L./mh-125	Jangli palak	Herb	Leaves	Paste	External	Wounds	0.14	0.33	23.81	41	0.64	0.59
				Roots	Paste	External	Skin problems						
**Primulaceae**													
85	*Androsace rotundifolia* Hardwicke/mh-128	Herb	Herb	Rhizome	Extract	Internal	Ophthalmic diseases	0.21	0.5	35.71	25	0.39	0.67
				Leaves	Infusion	Internal	Stomach problems, skin diseases						
**Punicacea**													
86	*Punica granatum* Linn./mh-129	Druna	Tree	Fruit	Eaten	Internal	Cough, tonic	0.5	0.83	66.67	52	0.81	1
				Leaves	Juice	Internal	Dysentery						
				Bark stem and root	Decoction	Internal	Anthelmintic, especially for tapeworms, mouthwash, expectorant						
**Pteridaceae**													
87	*Pteris cretica* L./mh-131	Fern	Fern	Leaves	Paste	External	Wounds	0.07	0.17	11.9	9	0.14	0.17
**Ranunculaceae**													
88	*Anemone tetrasepala* Royle/mh-132	Herb	Herb	Roots	Juice	External	Boils	0.07	0.17	11.9	12	0.19	0.34
89	*Aquilegia pubiflora* Wall ex Royle./mh-133	Herb	Herb	Root	Paste	External	Snake bite, emetic, toothache	0.36	0.5	42.86	37	0.58	0.45
				Flower	Paste	External	Skin burns, wound						
90	*Caltha alba* Camb. var. alba/mh-136	Herb	Herb	Aerial parts	Juice	Internal	Antispasmodic, sedative	0.14	0.33	23.81	29	0.45	0.28
91	*Clematis buchananiana* DC./mh-138	Langi	Shrub	Leaves	Paste	External	Skin infection, chambal wounds	0.36	0.5	42.86	43	0.67	0.75
				Roots	Crushing and wrapping	External	Bleeding from nose						
				Roots	Poultice	External	Swellings, inflammation						
				Roots	Juice	Internal	Peptic ulcers						
92	*Clematis montana* Buch./mh-139	Langi, shrub	Shrub	Leaves	Extract	Internal	Diabetes	0.14	0.33	23.81	27	0.42	0.33
				Flowers	Decoction	Internal	Cough						
93	*Ranunculus muricatus* L./mh-140	Herb	Herb	Aerial parts	Cooked	Internal	Asthma	0.07	0.17	11.9	14	0.22	0.19
**Rhamnaceae**													
94	*Ziziphus nummularia* (Burm.f.) Wight & Arn./mh-141	Ber	Tree	Fruit	Decoction	External	Dandruff	0.21	0.33	27.38	51	0.8	0.98
				Bark	Mixed with Milk and honey	Internal	Diarrhea and dysentery						
**Rosaceae**													
95	*Eriobotrya japonica* Thumb. Lindler/mh-142	Loquat	Tree	Leaves	Poultice	External	Swellings	0.36	0.5	42.86	44	0.69	0.89
				Fruits	Eaten	Internal	Sedative, vomiting						
				Leaves	Infusion	Internal	Relieve diarrhea						
				Flowers	Infusion	Internal	Tea						
96	*Prunus armeniaca* Linn./mh-144	Hari, khubani, apricot	Tree	Fruit	Eaten	Internal	Laxative	0.14	0.33	23.81	31	0.48	0.39
				Seed	Oil	External	Softening effect on the skin						
97	*Prunus domestica* Linn./mh-145	Lucha, Alu bukhara	Tree	Fruit	Eaten	Internal	Irregular menstruation, debility, miscarriage, used for alcoholic beverages and liqueurs	0.43	0.33	38.1	34	0.53	0.84
98	*Prunus persica* Linn. Batch/mh-146	Aru, peach	Tree	Leaves	Juice	Internal	Gastritis, whooping cough and bronchitis, kill intestinal worms, remove maggots from wounds in cattle and dogs	0.36	0.5	42.86	44	0.69	0.88
99	*Pyrus malus* L./mh-147	Saib	Tree	Fruit	Juice, paste	Internal	Rheumatism, hypertension, tonic for vigorous body, strengthen bones, face spots	0.36	0.5	42.86	46	0.72	0.81
100	*Pyrus pashia* Ham.ex D. Don/mh-148	Butangi	Tree	Fruit	Eaten	Internal	Dark circles around the eyes, constipation	0.14	0.33	23.81	49	0.77	0.95
101	*Rosa brunonii* Lindl./mh-151	Chal, tarni, musk rose	Shrub	Flower	Decoction	Internal	Constipation	0.5	0.83	66.67	57	0.89	0.98
				Flowers	Powder	Internal	Diarrhea, heart tonic, skin and eye diseases						
				Leaf	Juice	External	Cuts, wounds						
102	*Rubus fruticosus* Hk f. non L/mh-153	Garachey	Shrub	Leaves	Infusion	Internal	Diarrhea, fever	0.21	0.5	35.71	32	0.5	0.59
				Bark	Soaking	Internal	Diabetes						
103	*Rubus niveus* Thunb./mh-154	Garachey	Shrub	Leaves	Extract	External	Urticaria	0.5	0.67	58.33	41	0.64	0.69
				Leaves	Powder	Internal	Diarrhea, fever, and diuretic						
				Root	Decoction	Internal	Dysentery, colic pains, whooping coughs						
104	*Duchesnea indica* (Andrews) Teschem/mh-155	Budimewa	Herb	Fruit	Juice	Internal	Eye infection, tonic	0.14	0.33	23.81	33	0.52	0.61
105	*Fragaria nubicola* Lindl. ex Lacaita/mh-157	Budi meva, Wild Straberry	Herb	Fruit	Chewed	Internal	Laxative, purgative, mouth infection	0.21	0.33	27.38	35	0.55	0.5
**Rubicaceae**													
106	*Galium aparine* L./mh-158	Lainda	Herb	Aerial parts	Powder	External	Bleeding	0.07	0.17	11.9	15	0.23	0.31
107	*Galium asperifolium* Wall/mh-159	Lainda	Herb	Aerial parts	Juice	Internal	Diuretic, kidney infections	0.14	0.33	23.81	22	0.34	0.38
**Rutaceae**													
108	*Skimmia laureola* DC. Sieb/mh-161	Tree	Tree	Leaves	Powdered	External	Smallpox, worm problems, colic	0.21	0.5	35.71	48	0.75	0.59
109	*Zanthoxylum armatum* DC. Prodr/mh-162	Timbar	Shrub	Fruit, branches	Juice	Internal	Gas trouble, cholera, stomach disorder, piles, gum, toothache, indigestion	0.64	0.67	65.48	60	0.94	1.13
				Seed	Powder, chewed	Internal	Stomach problems, toothache						
**Salicaceae**													
110	*Salix acmophylla* Boiss./mh-164	Beens, bed, gaith	Tree	Leaves	Paste, boiled with *Robinia pseudoacacia* and *Cotula anthemoids*	Internal	Boils, hernia, fever and swelling of joints	0.36	0.67	51.19	51	0.8	0.98
				Branch	Chewing	Internal	Stomach problems						
111	*Salix denticulate* Andersson/mh-166	Beens	Tree	Stem and root bark	Boiled	Internal	Fever, headache and paralysis	0.29	0.67	47.62	34	0.53	0.39
				Leaves, branches	Paste	External	Itching and allergy						
**Sambucaceae**													
112	*Sambucus wightiana* Wall. ex Wight & Arn./mh-167	Gandala	Herb	Fruit	Eaten	Internal	Stomach problems, expel worms	0.14	0.33	23.81	19	0.3	0.5
**Sapindaceae**													
113	*Sapindus mukorossi* Gaertn./mh-168	Ritha, Soap nut	Tree	Seeds	Powdered	External	Insect killer	0.14	0.33	23.81	47	0.73	0.77
				Fruits	Rubbing	External	Burns						
**Saxifragaceae**													
114	*Bergenia ciliate* Haw. Sternb./mh-170	Zakhm-e-Hayat	Herb	Aerial parts	Powder	Internal	Urinary tract troubles	0.36	0.5	42.86	29	0.45	0.39
				Leaves	Juice	External	Earache						
				Root	Juice	Internal	Cough and cold, kidney stones						
**Scorphulariaceae**													
115	*Verbascum thapsus* L./mh-172	Gider tabacoo	Herb	Roots	Decoction	Internal	Toothache, cramps, convulsions	0.21	0.33	27.38	17	0.27	0.25
**Smilicaceae**													
116	*Smilax glaucophylla* Klotroch/mh-174	Epiphyte	Epiphyte	Aerial parts	Infusion	Internal	Flatulence, fever, dog bite and spasm	0.29	0.67	47.62	32	0.5	0.55
**Ulmaceae**													
117	*Celtis caucasica* Willd/mh-175	Batkaral	Tree	Aerial parts	Juice	Internal	Colic and amenorrhea	0.14	0.33	23.81	17	0.27	0.45
**Urticaceae**													
118	*Debregeasia salicifolia* D. Don Rendle/mh-178	Sandari	Shrub	Aerial parts	Paste	External	Skin rashes, dermatitis and eczema	0.21	0.17	19.05	15	0.23	0.41
**Valerianaceae**													
119	*Valeraina jatamansi* Joes./mh-179	Herb	Herb	Aerial parts	Oil	Internal	Constipation	0.07	0.17	11.9	19	0.3	0.41
**Violaceae**													
120	*Viola canescens* Wall.ex Roxb./mh-181	Banafsha	Herb	Leaves	Juice	Internal	Cough, cold, fever, jaundice	0.29	0.5	39.29	51	0.8	0.84
121	*Viola pilosa* Blume./mh-182	Banafsha	Herb	Leaves	Decoction	Internal	Pain, fever, stomach ulcer	0.21	0.5	35.71	47	0.73	0.81

**Key words**: **Rel BS** = Relative number of body system treated by a single species; **Rel PH** = Relative number of pharmacological properties for a single plant; **RI** = Relative importance, **FC** = Frequency of citation; **RFC** = relative frequency of citation; **UV** = Use Value

**Table 4:** Informant consensus factor for different disease categories.

	Disease Categories	Symptoms	Ntax	Nur	Fic	Most Commonly Used Plants
1	**Musculoskeletal and nervous system**	Nervous problem, weakness, muscular pains, sedative, cramps, colic, depression, paralysis	22	197	0.89	*Hypericum perforatum*, *Juglans regia*, *Pyrus malus*, *Heracleum cachemirica*, *Heracleum candicans*,
2	**Gastro-intestinal, parasitic and hepatobiliary**	Liver and bile diseases, jaundice, vomiting, dyspepsia, hepatic pain, dysentery, loss of appetite, anthelmintic, improve digestion, nausea, piles, intestinal parasites, stomach ache, constipation, flatulence, diarrhea, hernia, cholera, gas trouble	114	1162	0.90	*Mentha royleana*, *Zanthoxylum armatum*, *Berberis lycium*, *Eriobotrya japonica*, *Punica granatum*, *Ziziphus numelaria*, *Artemisia absinthium*
3	**External injuries, bleeding**	Swellings, wounds, rheumatism, nail wound, inflammations, Joints pain, pain, burns, cuts and wounds, body inflammation, bone fracture, boils, burns, back pain, bleeding	65	552	0.88	*Hypericum perforatum*, *Berberis lycium*, *Sapindus mukorossi*, *Adiantum venustum*, *Rumex dentatus*
4	**Urinogenital and venereal**	Urinary problems, menorrhagia, miscarriage, abortion, amenorrhea, irregular menstruation, leucorrhoea, kidney stones, gonorrhea, contraceptive, debility	16	91	0.83	*Aesculus indica*, *Prunus domestica*, *Bergenia ciliata*, *Galium asperifolium*, *Oenothera rosea*, *Eriobotrya japonica*,
5	**Blood and lymphatic system**	Anemia, Hypertension, blood purifier.	15	76	0.81	*Dalbergia sissoo*, *Rosa brunonii*, *Berberis lycium*, *Vibernum nervosum*,
6	**Cardiovascular disease**	Heart tonic	6	25	0.79	*Rosa brunonii*, *Oenothera rosea*, *Viola canscens*, *Adiantum capillus-veneris*
7	**Pulmonary disease**	Respiratory problem, cough, difficult breathing, diseases of the lungs, chest pain, asthma, bronchitis, Flue	41	236	0.83	*Mentha royleana*, *Polygonatum multiflorum*, *Punica granatum*, *Pyrus pashia*, *Salvia moorcroftiana*, *Prunella vulgaris*
8	**Dermatological**	Skin problems, scabies, leukoderma, smallpox, warts, ulcers, urticaria, pimples, itching and allergy, freckles, cracked heels, measles, leprosy, dark circles around the eyes	47	306	0.85	*Fumaria indica*, *Adiantum incisum*, *Euphorbia wallichii*, *Gallium asperifolium*, *Rosa brunonii*
9	**Oral, dental, Hair and ENT**	Toothache, strengthen spongy gums, mouth infection, eye sight weakness, earache, flue and cough, sore throats, gum infection, pyorrhea, dandruff, hair tonic, headache	38	186	0.80	*Rosa brunonii*, *Androsace rotundifolia*, *Bergenia ciliata*
10	**Other (fever, tonic, cold, tumors)**	Tonic, sun burns, tumors, typhoid, fevers, colds, tumors, cooling agent, demulcent laxative, soft drinks.	45	258	0.83	*Fumaria indica*, *Adiantum incisum*, *Asparagus filicinus*, *Castanea sativa*, *Viola canscens*, *Trichodesma indicum*, *Punica granatum*, *Berberis lycium*, *Lagustrum lucidam*
11	**Antidote**	Snake bite, scorpion sting, dog bite	8	31	0.77	*Nerium oleander*, *Dioscorea deltoidea*, *Hypericum perforatum*
12	**Insectiside**	Anti lice, antiseptic, helminthiasis	9	27	0.69	*Juglans regia*, *Poa nepalensis*, *Desmodium polycarpum*
13	**Diabetes**	Diabetes	6	36	0.86	*Berberis lycium*, *Clematis montana*, *Rubus fruticosus*,

### Plant part(s) used

**Fig 3:**
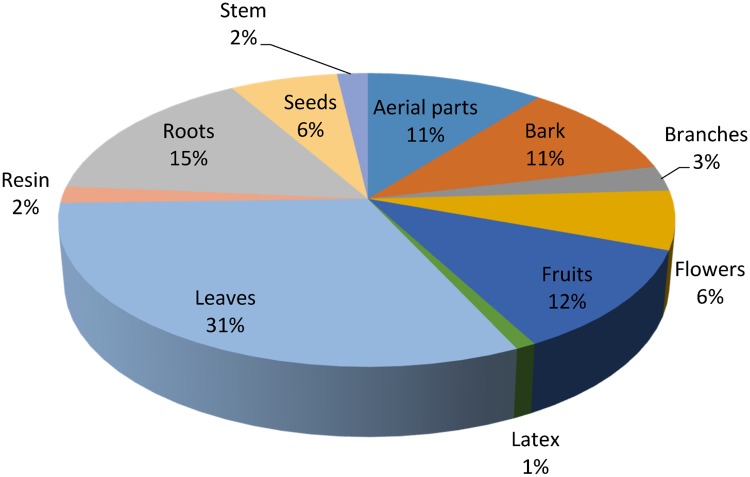
Plant parts used in herbal recipes.

Different plant parts are used differently in herbal medicines depending upon the cultural knowledge and availability of those parts to local inhabitants. In the present study, leaves (31%) were the most commonly used plant part utilized in herbal preparations followed by roots (15%), fruits (12%), bark and other aerial parts (11% each), and flowers and seeds (6% each) (Fig 3). Leaves are frequently used in herbal preparations due to their active secondary constituents. It is thought that leaves contain more easily extractable phytochemicals, crude drugs and many other mixtures which may be proven as valuable in phytotherapy [5, 50–51]. This may be the reason for several studies, including this one, reporting leaves as the most highly exploited plant part for medicinal uses [26, 52]. Besides leaves, roots are also favored parts in many cases possibly because they also contain higher concentrations of bioactive compounds than other plant parts [53–56]. In a few cases, the same plant parts are used to treat different diseases, for example, the roots of *Berberis lycium* are used internally for the treatment of chronic diarrhea, piles, diabetes, pustules and scabies while externally they are used to cure fractured bones and swellings. Similar uses of many other plants were also recorded (presented in Table 3).

### Method of preparation and administration

**Fig 4:**
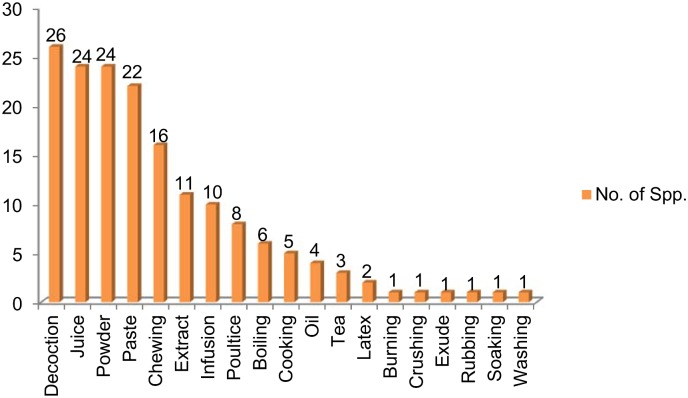
Methods of prepration of herbal recipes.

**Fig 5:**
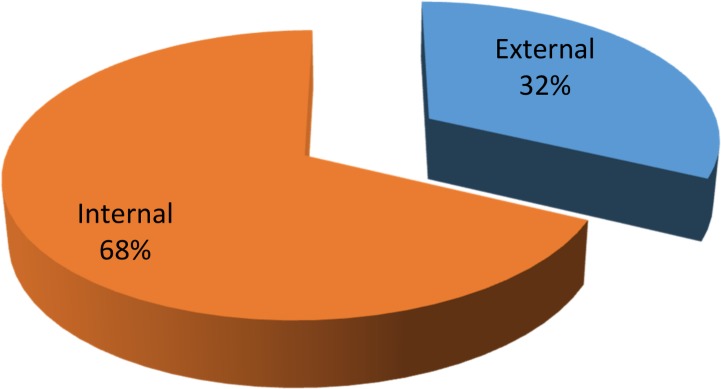
Mode of application of folk recipes.

The various plant parts were mostly used in decoctions (26 species) during herbal preparations, followed by juice and powder (24 species each), paste (22), chewing (16 species), extract (11 species), infusion (10 species) and poultice (8 species) (Fig 4), while considering the method of preparation and administration of herbal medicines, reports included decoction, paste, juice, powder or freshly taken. Decoctions are often found to be one of the major forms of preparation in ethnobotanical practice as they are easy to prepare by mixing with water, tea or soup [57]. The most frequent use of decoction might also be due to the fact that heating can cause acceleration of biological reactions resulting in the increased availability of many active compound [58–60]. Similar findings have also been reported by other studies. For example, among major forms of preparation in Madhupur forest area, Bangladesh, decoction was the most frequent (33%), followed by juice (24%), paste (18%), fruit (8%), oil (6%), vegetable (4%), latex (2%), powder (2%) and others (3%) [61]. Similar results are reported also from other parts of the world. Nondo *et al*. [62], for example, reported medicinal plants to treat malaria in the Kagera and Lindi regions of Tanzania. Among 108 plants most were taken orally or in the form of a decoction. Similarly Siew *et al*. [63] reported decoction as the main preparation method while documenting traditional uses of 104 plants from Singapore. The quantity and dosage of medicinal drugs is not fixed and differs with age, state of health of the patient and the severity of the disease. Most of the plants were used on their own, but in some herbal preparations specific plant parts were mixed with other ingredients in order to treat an ailment, including milk, honey, oil or butter. A few species were used in combination with other herbs, for example, the leaves of *Salix acmophylla* were boiled with *Robinia pseudoacacia* and *Cotula anthemoids* to treat fever and hernia. Most of the herbal preparations were taken internally (68%) with a smaller number used externally (32%) (Fig 5).

### Informant consensus factor

The Informant consensus factor (Fic) depends upon the availability of plants within the study area to treat diseases. In the present study, the Fic values ranged from 0.90 to 0.69 with an average of 0.82 which reflects a high consensus among the informants about the use of plants to treat ailments. The ailments are classified into 13 different categories and the maximum Fic value is for gastro-intestinal, parasitic and hepatobiliary complaints and the most cited plants used under this category are *Mentha royleana*, *Zanthoxylum armatum*, *Berberis lycium*, *Eriobotrya japonica*, *Punica granatum*, *Ziziphus numelaria* and *Artemisia absinthium*. A plant with insecticidal properties has the lowest Fic value of 0.69 which indicates that there is less awareness of people in the study area to use plants as insecticides (Table 4). Gastro-intestinal disorders were prevalent in the study area which can be attributed to limited availability of hygienic food and drinking water [64–65]. The plants frequently used to treat these disorders might contain active ingredients and thus were well known by locals. Among various classes of indigenous uses across the globe, various types of gastrointestinal disorders are predominant and a significant number of plant species have been discovered to cure such illnesses across different ethnic communities [66–67]. Ethnopharmaecological studies have shown that in some parts of the world, gastrointestinal disorder is a first use category [37, 42, 68–70]. A high Fic for gastrointestinal disorders has also been reported by other studies [9, 71–72] although there had previously been no study conducted in our study region. Our findings generally agree with previous results [16, 19, 46] while particularly supporting the results of Bibi *et al*. [73] who reported that digestive problems were the dominant diseases in the Mastung district of Balochistan, Pakistan.

The high ICF values obtained in this study indicate a reasonably high reliability of informants on the uses of medicinal plant species [74], particularly for gastrointestinal complaints, while low ICF values for cardiovascular diseases and antidotes indicate less uniformity of informants' knowledge. Frequently, a high ICF value is allied with a few specific plants with high use reports for treating a single disease category [75], while low values are associated with many plant species with an almost equal or high use reports suggesting a lower level of agreement among the informants on the use of these plant species to treat a particular disease category.

### Relative frequency of citation and use value

The RFC shows the local importance of every species with reference to the informants who cited uses of these plant species [76]. In our work, RFC ranges from 0.94 to 0.14 (Table 3). *Berberis lycium*, *Ajuga bracteosa*, *Prunella vulgaris*, *Adiantum capillus-veneris*, *Desmodium polycarpum*, *Pinus roxburgii*, *Albizia lebbeck*, *Cedrella serrata*, *Rosa brunonii*, *Punica granatum*, *Jasminum mesnyi* and *Zanthoxylum armatum* were the most cited ethnomedicinal plant species. These plants are dominant in the study area and the people are, therefore, very familiar with them. Moreover, these species are native to the area and have been known to local cultures over a long time period. Thus their specific properties for curing different diseases have become popularized and well-established among the indigenous people. These results are important as they could form an important research baseline for subsequent evaluation of plant-derived medicinal compounds, potentially resulting in future drug discoveries [77]. The plant species having high RFC values should be subjected to pharmacological, phytochemical and biological studies to evaluate and prove their authenticity for development of marketable products [78]. These species should also be prioritized for conservation as their preferred uses may place their populations under threat due to over harvesting.

The use value (UV) is a measure of the types of uses attributed to a particular plant species. In the present study *Berberis lyceum*, *Ajuga bracteosa*, *Abies pindrow*, *Prunella vulgaris*, *Adiantum capillus-veneris*, *Desmodium polycarpum* and *Pinus roxburgii* were ascribed UV values of 1.13, 1.13, 1.03, 1.00, 1.00, 0.98, and 0.98 respectively. UV determines the extent to which a species can be used; thus species with a high UV are more exploited in the study area to cure a particular ailment than those with a low UV. It is found that plants having more use reports (UR) always have high UVs while those plants having fewer URs reported by informants have lower UV. It is also observed that plants which are used in some repetitive manner are more likely to be biologically active [79].

As the values for the UV and RFC are dynamic and change with location and with the knowledge of the people, so the values of UV and RFC may vary from area to area and even within the same area. Plants with lower UV and RFC values are not necessarily unimportant, but their low values may indicate that the young people of the area are not aware about the uses of these plants and, therefore that the understanding of their use is at risk of not being transmitted to future generations, thus this knowledge may eventually disappear [80].

This was the first quantitative ethnobotanical investigation to be carried out in the study area; therefore we compared our results with similar quantitative studies carried out in other parts of the country [26, 50, 51]. This revealed that there were differences in most of the cited species and their quantitative values. In a study carried out by Abbasi *et al*. [26], *Ficus carica* and *Ficus palmata* were the most cited species, while Bano *et al*. [51] reported that *Hippophae rhamnoides* had the highest use value (1.64) followed by *Rosa brunonii* (1.47). These differences can be mostly likely accounted for by variations in the vegetation and geo-climate of the study areas and emphasizes the need for more quantitative studies in a wider range of locations, but particularly in the more remote, mountainous regions where there is still a strong reservoir of ethnomedicinal knowledge amongst the indigenous communities.

### Relative importance

The species with high RI values are highly versatile and used to treat a number of diseases. The highest RI values were obtained for *Berberis lyceum*, *Ajuga bracteosa*, *Prunella vulgaris*, *Adiantum capillus-veneris*, *Desmodium polycarpum*, *Pinus roxburgii*, *Albizia lebbeck*, *Cedrella serrata* and *Rosa brunonii*, indicating that these plants are widely used in the study area. These plants have high RI values because they are used in treating various body systems, i.e. local people have considerable knowledge about these plants. The importance of a plant increases as it is used to treat more infirmities [81].

### Jaccard index (Novelty index)

Due to differences in their origins and cultures, indigenous communities differ greatly in their ethno-botanical knowledge. Documenting and comparing this knowledge can reveal the considerable depth of knowledge among communities which can result in novel sources of drug development [82]. Such studies also point out the importance of indigenous knowledge on medicinal plants, with differences between regions arising as a result of historical [83], ecological [84], phytochemical and even organoleptic [85] differences. The results of the present study were compared with those from twelve national and international studies conducted in areas similar in terms of their cultural values and climatic conditions to the study area (Table 5). The data show that across 121 plant species, the similarity percentage ranges 16.5 from 0 while the dissimilarity percentage ranges from 22.5 to 1.05. The highest degree of similarity index was with studies by Khan *et al*. 2010 [86], Amjad *et al*. 2015 [30], Ahmed *et al*. 2013 [87] and Shaheen *et al*. 2012 [88] with JI values of 32.88, 26.19, 19.12, 18.70 respectively. These studies are all from areas in the vicinity of the study area where ethnic values, historical and ecological factors are similar. In addition, there are similar vegetation types and it is also possible that cross cultural exchange of knowledge could have occurred between indigenous communities, either recently or in the past, which also might provide a reason for the high similarity index values. The lowest JI values were for the studies conducted by Kichu *et al*. 2015 [89] and Bahar *et al*. 2013 [90]. These studies were carried out at a greater distance from our study location, and thereby reflect a greater difference in ethno-botanical knowledge due to differences in population size, species diversity and habitat structure. Furthermore there would be less chance of the exchange of cultural knowledge between the areas were these studies were conducted and our study location as the areas are isolated by mountain ranges and cultural variations. These findings are in agreement with studies carried out by Kyani and coworker [91] and Ijaz and his coworker [29]. This comparative analysis strengthens the value of the ethnobotanical knowledge from our study location by emphasizing the novelty of our findings, whilst also providing a basis for future studies.

**Table 5:** Jaccard index comparing the present study with previous reports at regional, national and global scales.

Area	Study year	Number of recorded plant species	Plants with similar use	Plants with dissimilar use	Total species common in both area	Species enlisted only in aligned areas	Species enlisted only in study area	% of plant with similar uses	% of dissimilar uses	JI	Citation
Poonch Valley, Azad Kashmir, Pakistan	2010	169	28	20	48	121	73	16.6	11.8	32.9	[86]
Pir Nasoora National Park Azad Kashmir, Pakistan	2015	104	10	23	33	71	88	9.62	22.1	26.2	[12]
30Bana Valley, Azad Kashmir, Pakistan	2015	86	5	15	20	66	101	5.81	17.4	13.6	[92]
Bagh, Azad Kashmir, Pakistan	2012	71	7	16	23	48	98	9.86	22.5	18.7	[88]
Neelum valley, Azad Kashmir, Pakistan	2011	40	2	5	7	33	114	5	12.5	5	[93]
Leepa valley, Azad Kashmir Pakistan	2012	36	4	3	7	29	114	11.1	8.33	5.15	[94]
Patriata, New Muree, Pakistan	2013	93	8	18	26	67	95	8.6	19.4	19.1	[87]
Abbottabad, KPK, Pakistan	2016	74	6	8	14	60	107	8.11	10.8	9.15	[26]
Alpine and Subalpine region of Pakistan	2015	125	6	11	17	108	104	4.8	8.8	8.72	[22]
Naran valley, Paksitan	2013	101	9	18	27	74	94	8.91	14.87	13.85	[95]
Nagaland, India	2015	135	0	3	3	132	118	0	2.22	1.21	[89]
Madonie Regional Park, Italy	2013	174	0	3	3	171	118	0	1.72	1.05	[11]
Marmaris, Turkey	2013	64	0	3	3	61	118	0	4.69	1.7	[90]

### Statistical analysis

**Fig 6:**
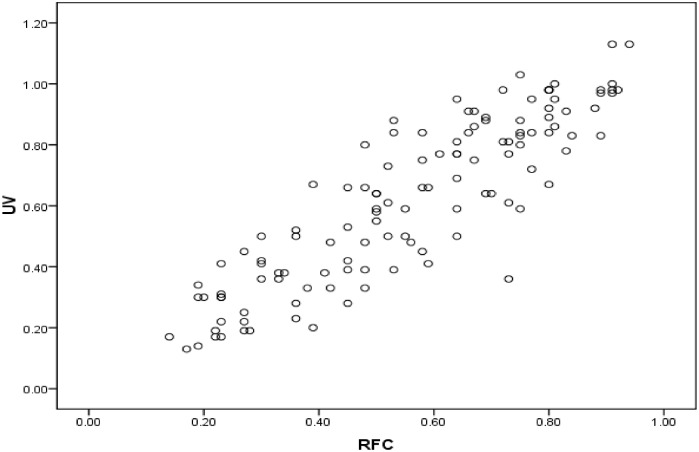
Association between use value and relative frequency of citation.

The Pearson correlation coefficient between UV and RFC is 0.881 which reflects that there is a significant and positive correlation between the proportion of uses of a plant species within a sample of interviewed people and the number of times that a particular use of a species is mentioned by the informant (Table 6). This shows that with an increase in the number of informants the knowledge of the uses of a particular species also increases. These results indicate that the study can make a significant contribution to folk knowledge on the use of medicinal plants and further laboratory-based investigations could help in identifying the active ingredients of the most commonly exploited plants. The coefficient of determination defined as r^2^ determines the degree of variation among the data. In the present study the value of R^2^ is 0.77 which means that 77% of the variability in UV can be explained in terms of the RFC [25, 59]. Fig 6 illustrates the positive correlation between the values of RFC and UV.

**Table 6:** Relationship between Use value (UV) and Relative frequency of citation (RFC).

	Correlations	UV	RFC
UV	Pearson Correlation	1	.881**
	Sig. (2-tailed)		.000
	N	121	121
RFC	Pearson Correlation	.881**	1
	Sig. (2-tailed)	.000	
	N	121	121

**. Correlation is significant at the 0.01 level (2 -tailed).

R^2^ = 0.77

## Conclusions

This paper reviews 121 species which are identified as being exploited by local people for their recognized importance in indigenous health care in the Toli Peer National Park. The most common plants in the study area with an ethnomedicinal value are *Berberis lycium*, *Ajuga bracteosa*, *Prunella vulgaris*, *Adiantum capillus-veneris*, *Desmodium polycarpum*, *Pinus roxburgii*, *Albizia lebbeck*, *Cedrella serrata*, *Rosa brunonii*, *Punica granatum*, *Jasminum mesnyi* and *Zanthoxylum armatum*, all of which have high UV, RFC and relative importance values. The Pearson correlation coefficient between UV and RFC is 0.881, with a p value <1, which reflects a significant positive correlation between the use value and relative frequency of citation. The coefficient of determination value is 0.77 which means that 77% of the variability in the UV can be explained in terms of the RFC. The wild plant diversity in this remote National Park provides an effective and cheap source of health care for the local people. The plants employed in their indigenous herbal preparations could have great potential and should be subject to pharmacological screening, chemical analysis for bioactive ingredients and potential formulation as standard drug preparations to cure a range of ailments. The flora of the National Park is currently threatened by overgrazing, deforestation, and soil erosion which are the main causes of reduction of medicinal and other plants in the area. It is therefore essential to have a conservation strategy for the flora of the National Park, with special emphasis on species that are valued as medicinal plants.

## Supporting information

S1 File Interview guidelines followed during conducting field survey for obtaining ethnobotanical information. (DOCX)

S2 File Sample of Questionnaire used during field survey for obtaining ethnobotanical information. (DOCX)
