# Correction: Descriptive study of plant resources in the context of the ethnomedicinal relevance of indigenous flora: A case study from Toli Peer National Park, Azad Jammu and Kashmir, Pakistan

**DOI:** 10.1371/journal.pone.0180917

**Published:** 2017-07-03

**Authors:** Muhammad Shoaib Amjad, Mirza Faisal Qaseem, Israr Ahmad, Sami Ullah Khan, Sunbal Khalil Chaudhari, Nafeesa Zahid Malik, Humaira Shaheen, Arshad Mahmood Khan

The second author’s name is incorrect. The correct name is: Mirza Faisal Qaseem. The last author’s name is incorrect. The correct name is: Arshad Mahmood Khan. The correct citation is: Amjad MS, Qaseem MF, Ahmad I, Khan SU, Chaudhari SK, Zahid Malik N, et al. (2017) Descriptive study of plant resources in the context of the ethnomedicinal relevance of indigenous flora: A case study from Toli Peer National Park, Azad Jammu and Kashmir, Pakistan. PLoS ONE 12(2): e0171896. https://doi.org/10.1371/journal.pone.0171896.

In the author contributions, Muhammad Shoaib Amjad (MSA) should be listed as one of the persons who contributed to Conceptualization, Data curation, Formal analysis, Funding acquisition, Investigation, Methodology, Project administration, Resources, Visualization and Writing–review & editing.

There is an error in the last sentence under the subheading “Collection and identification of plants” in the Materials and methods section. The correct sentence is: The fully determined vouchers were deposited in the herbarium of the Department of Botany, Women University of Azad Jammu & Kashmir, Bagh, Pakistan.

## References

[1] Amjad MS, Qaeem Mf, Ahmad I, Khan SU, Chaudhari SK, Zahid Malik N, et al (2017) Descriptive study of plant resources in the context of the ethnomedicinal relevance of indigenous flora: A case study from Toli Peer National Park, Azad Jammu and Kashmir, Pakistan. PLoS ONE 12(2): e0171896 doi:10.1371/journal.pone.0171896 28192466 10.1371/journal.pone.0171896 PMC5305106

