# Supplementary material for: Descriptive study of plant resources in the context of the ethnomedicinal relevance of indigenous flora: A case study from Toli Peer National Park, Azad Jammu and Kashmir, Pakistan
Source: PLoS One. 2017 Feb 13;12(2):e0171896. doi: 10.1371/journal.pone.0171896 (PMC5305106; doi:10.1371/journal.pone.0171896)
Supplement: S1 File — (DOCX) [file pone.0171896.s001.docx]

**Interview Guideline**

**For conducting survey the guidline provided by the international society of ethnobiology was followed**

*The detatils are available on:* <http://ethnobiology.net/code-of-ethics/>

**Code of Ethics**

The Code of Ethics of the International Society of Ethnobiology (ISE) reflects the vision

of the Society and provides a framework for decisionmaking

and conduct for ethnobiological research and related activities. The goals are to facilitate ethical conduct and equitable relationships, and foster a commitment to meaningful collaboration and reciprocal responsibility by all parties. The Code of Ethics is a living document that will adapt over time to meet changing understandings and circumstances. All Members of the ISE are bound in good faith to abide by the Code of Ethics as a condition of membership.

The Code of Ethics is comprised a Preamble, Purpose, 17 Principles, 12 Practical

Guidelines and a Glossary of Terms. The Principles include:

· Prior Rights and Responsibilities

· SelfDetermination

· Inalienability

· Traditional Guardianship

· Active Participation

· Full Disclosure

· Educated Prior Informed Consent

· Confidentiality

· Respect

· Active Protection

· Precaution

· Reciprocity, Mutual Benefit and Equitable Sharing

· Supporting Indigenous Research

· The Dynamic Interactive Cycle

· Remedial Action

· Acknowledgement and Due Credit

· Diligence

The fundamental value underlying the Code of Ethics is the concept of ***mindfulness*** – a continual willingness to evaluate one's own understandings, actions, and responsibilities to others. The Code of Ethics acknowledges that biological and cultural harms have resulted from research undertaken without the consent of Indigenous peoples. It affirms the commitment of the ISE to work collaboratively, in ways that: support community driven development of Indigenous peoples’ cultures and languages; acknowledge Indigenous cultural and intellectual property rights; protect the inextricable linkages between cultural, linguistic and biological diversity; and contribute to positive, beneficial and harmonious relationships in the field of ethnobiology. The Code of Ethics applies to all research, collections, databases, publications, images, audio or video recordings, or other products of research and related activities undertaken, specially that which concerns collation and use of traditional knowledge or collections

of flora, fauna, or other elements of biocultural heritage found on community lands or

territories. The Principles and Practical Guidelines are based on the concept of traditional resource rights. They facilitate compliance with the standards set by national and international law and policy and customary practice. They recognize traditional and customary laws, protocols, and methodologies extant within the communities where collaborative research

is proposed. They are intended to support and enable but not override community level processes and decisionmaking structures, recognizing that Indigenous, traditional or local peoples conducting research within their own communities, for their own uses, may need to comply with their own cultural protocols and practices. In the event of inconsistency between such local requirements and the ISE Code of Ethics, all parties involved are encouraged to work collaboratively to develop appropriate practices.
