# Supplementary material for: Descriptive study of plant resources in the context of the ethnomedicinal relevance of indigenous flora: A case study from Toli Peer National Park, Azad Jammu and Kashmir, Pakistan
Source: PLoS One. 2017 Feb 13;12(2):e0171896. doi: 10.1371/journal.pone.0171896 (PMC5305106; doi:10.1371/journal.pone.0171896)
Supplement: S2 File — (DOCX) [file pone.0171896.s002.docx]

**Qquestionnaire used for collection of ethnomedicinal informations**

**Informant detail:**

Name:__________, Gender:_____________ Age:_______________ Education level:_________ Profession:__________________, Tribe:_______________

How long you are living in the area:________________________________

**Informants consent for the participation in the study:**

I ______________________ hereby give my full consent and conscious to participate in this study and declare that to the best of my Knowledge the information that i have provided are true, accurate and complete.

Signature/Thumb impression of informants:________________________Date:_____________

**Ethnomedicinal information:**

| **Botanical Name** | **Local Name** | | **Life form** |  | **Medicinal Uses** | | | |
| --- | --- | --- | --- | --- | --- | --- | --- | --- |
|  |  |  |  |  | **Uses** | **Part used** | **Recipes** | **Mode of administration** |
|  | |  |  |  |  |  |  |  |
|  | |  |  |  |  |  |  |  |
|  | |  |  |  |  |  |  |  |
|  | |  |  |  |  |  |  |  |
|  | |  |  |  |  |  |  |  |
|  | |  |  |  |  |  |  |  |
|  | |  |  |  |  |  |  |  |
|  | |  |  |  |  |  |  |  |
|  | |  |  |  |  |  |  |  |
|  | |  |  |  |  |  |  |  |
|  | |  |  |  |  |  |  |  |
|  | |  |  |  |  |  |  |  |
|  | |  |  |  |  |  |  |  |
